# Supplementary figures and images for: HIV proviral genetic diversity, compartmentalization and inferred dynamics in lung and blood during long-term suppressive antiretroviral therapy
Source: PLoS Pathog. 2022 Nov 4;18(11):e1010613. doi: 10.1371/journal.ppat.1010613 (PMC9668181; doi:10.1371/journal.ppat.1010613)

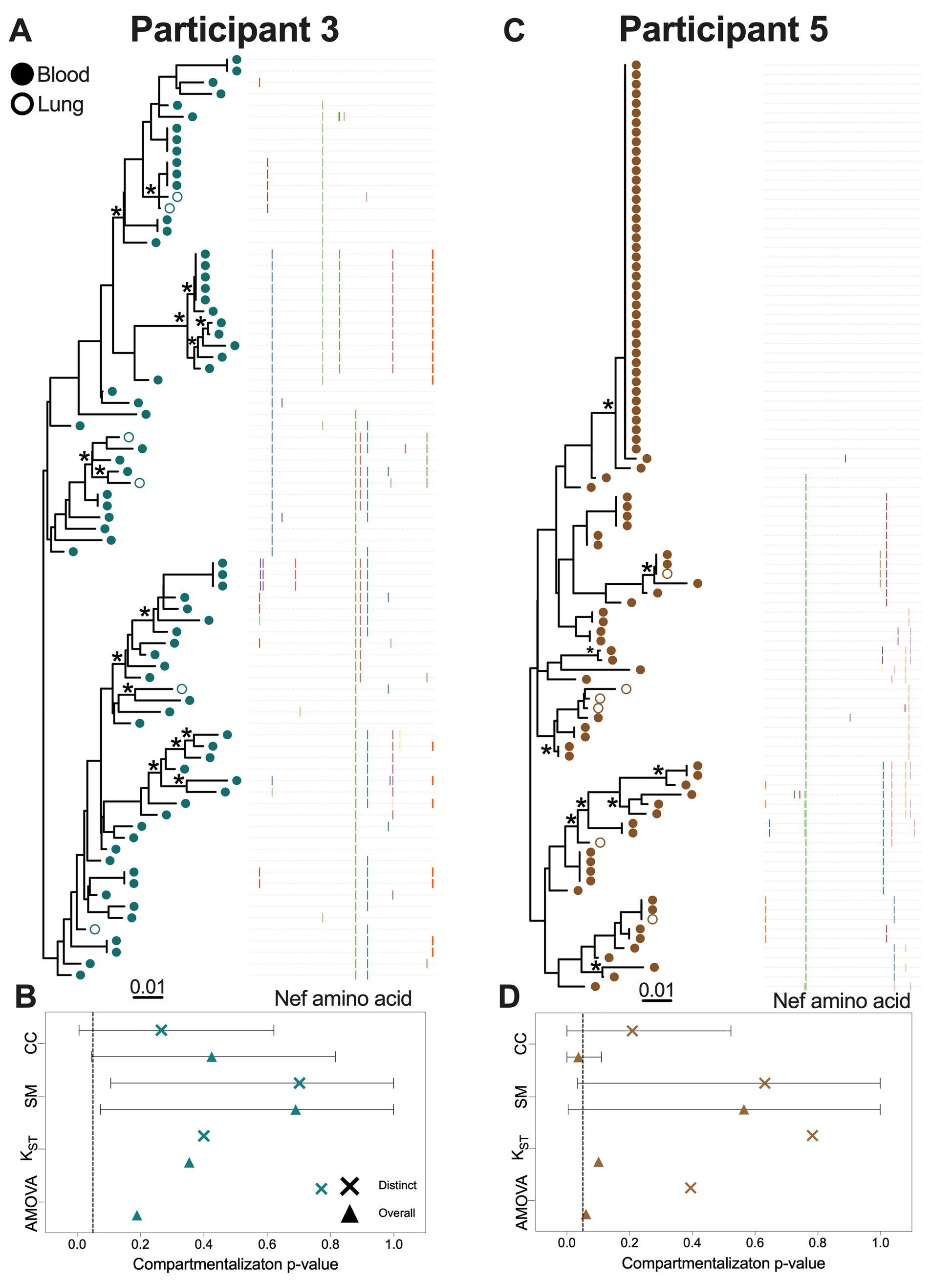

Supplement: S1 Fig — Legend as in Fig 2. (TIF) [file ppat.1010613.s001.tif]

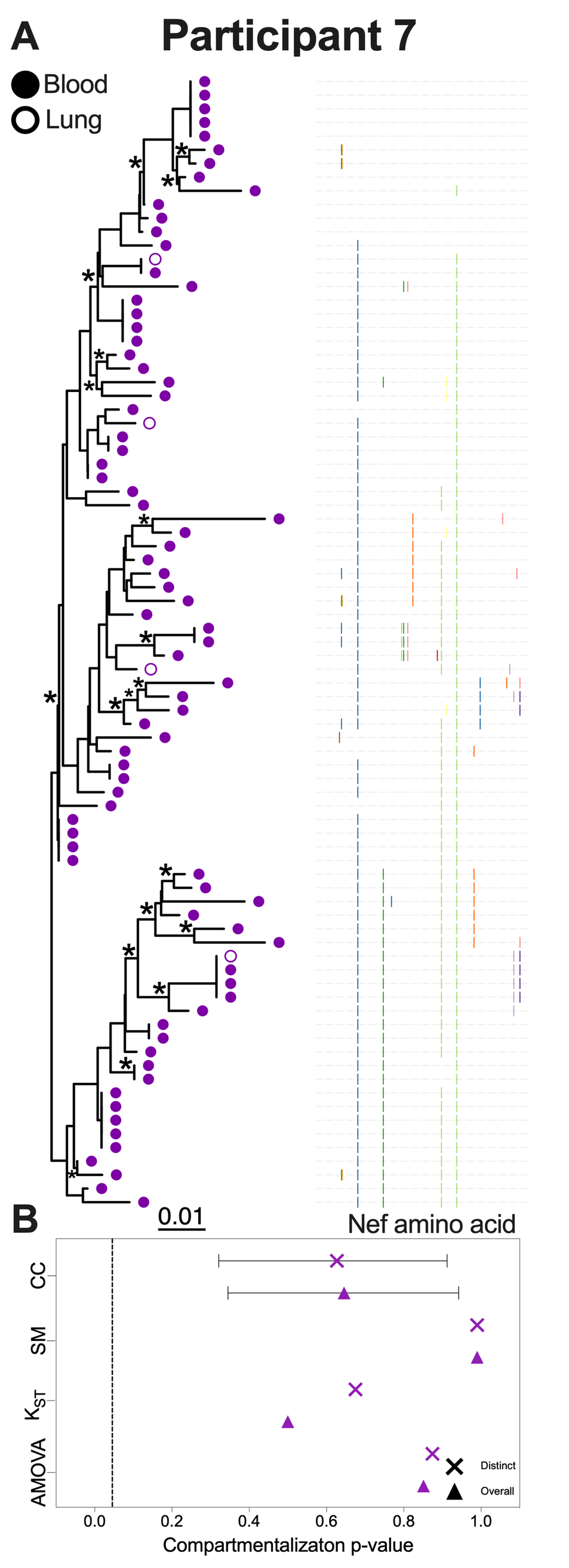

Supplement: S2 Fig — Legend as in Fig 2. (TIF) [file ppat.1010613.s002.tif]

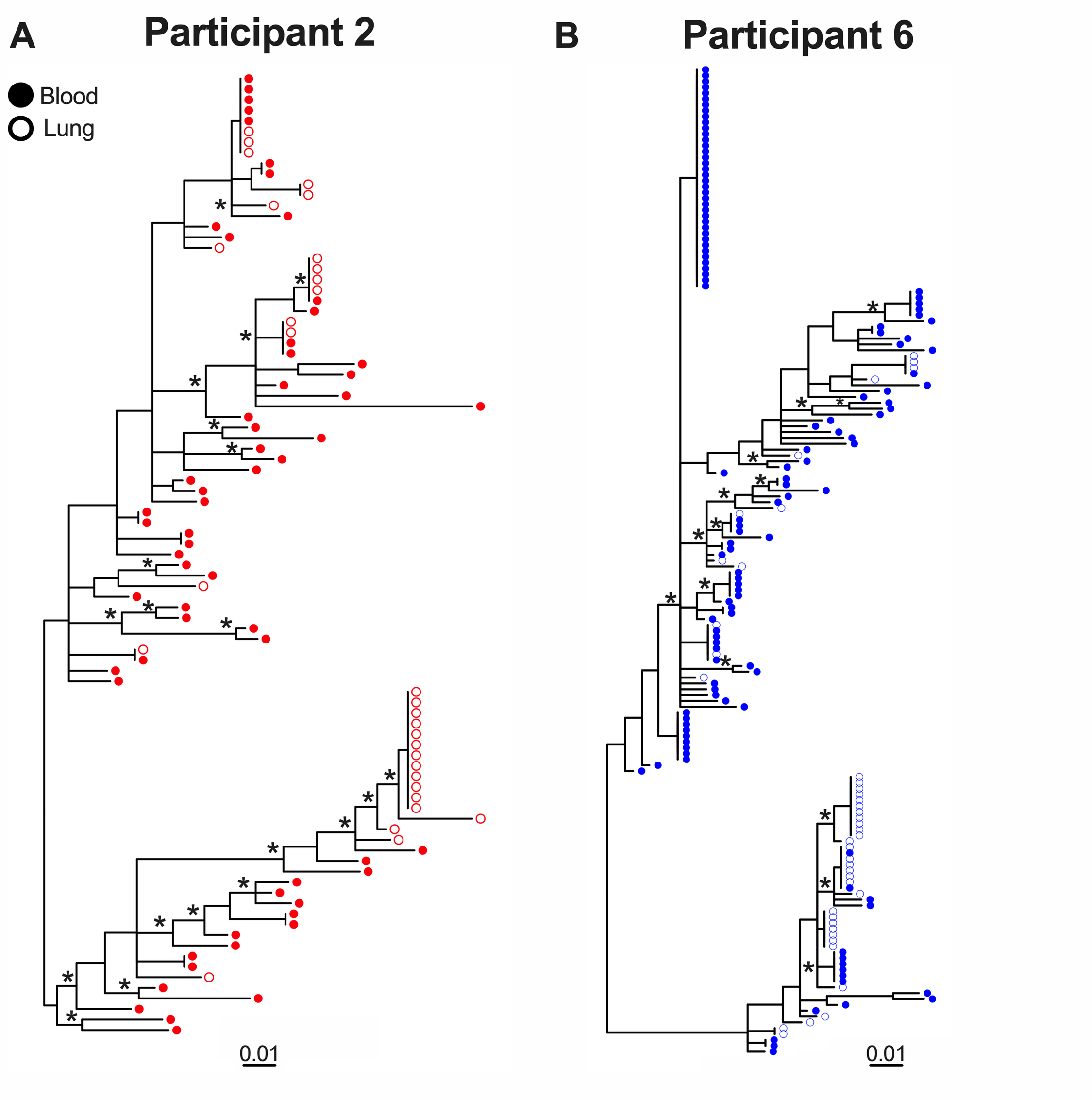

Supplement: S3 Fig — Consensus phylogeny derived from Bayesian inference of 7,500 trees per participant, rooted at the midpoint. Filled circles denote blood sequences; open circles denote lung sequences. Asterisks identify nodes supported by posterior probabilities ≥70%. (TIF) [file ppat.1010613.s003.tif]

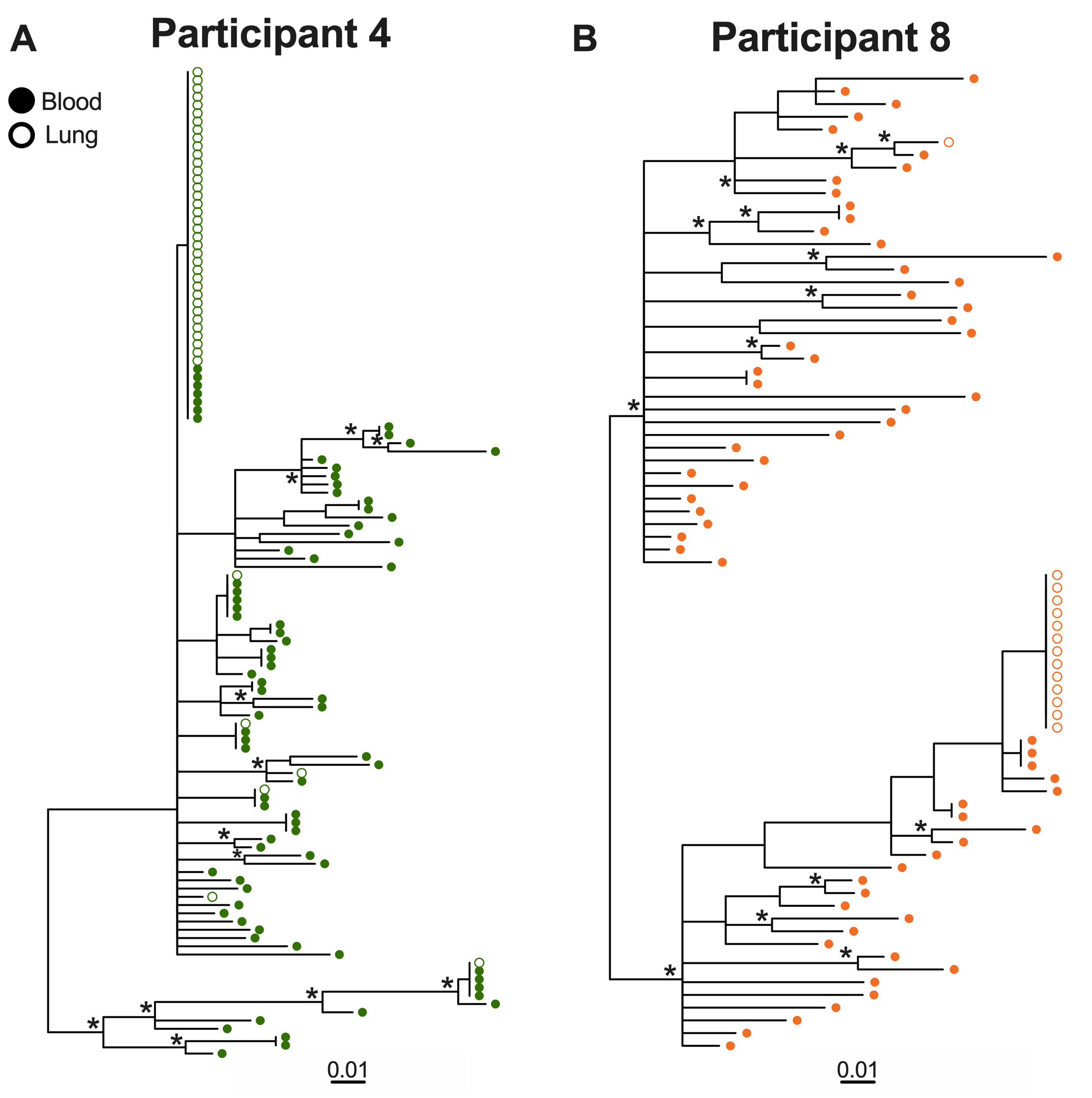

Supplement: S4 Fig — Legend as in S3 Fig. (TIF) [file ppat.1010613.s004.tif]

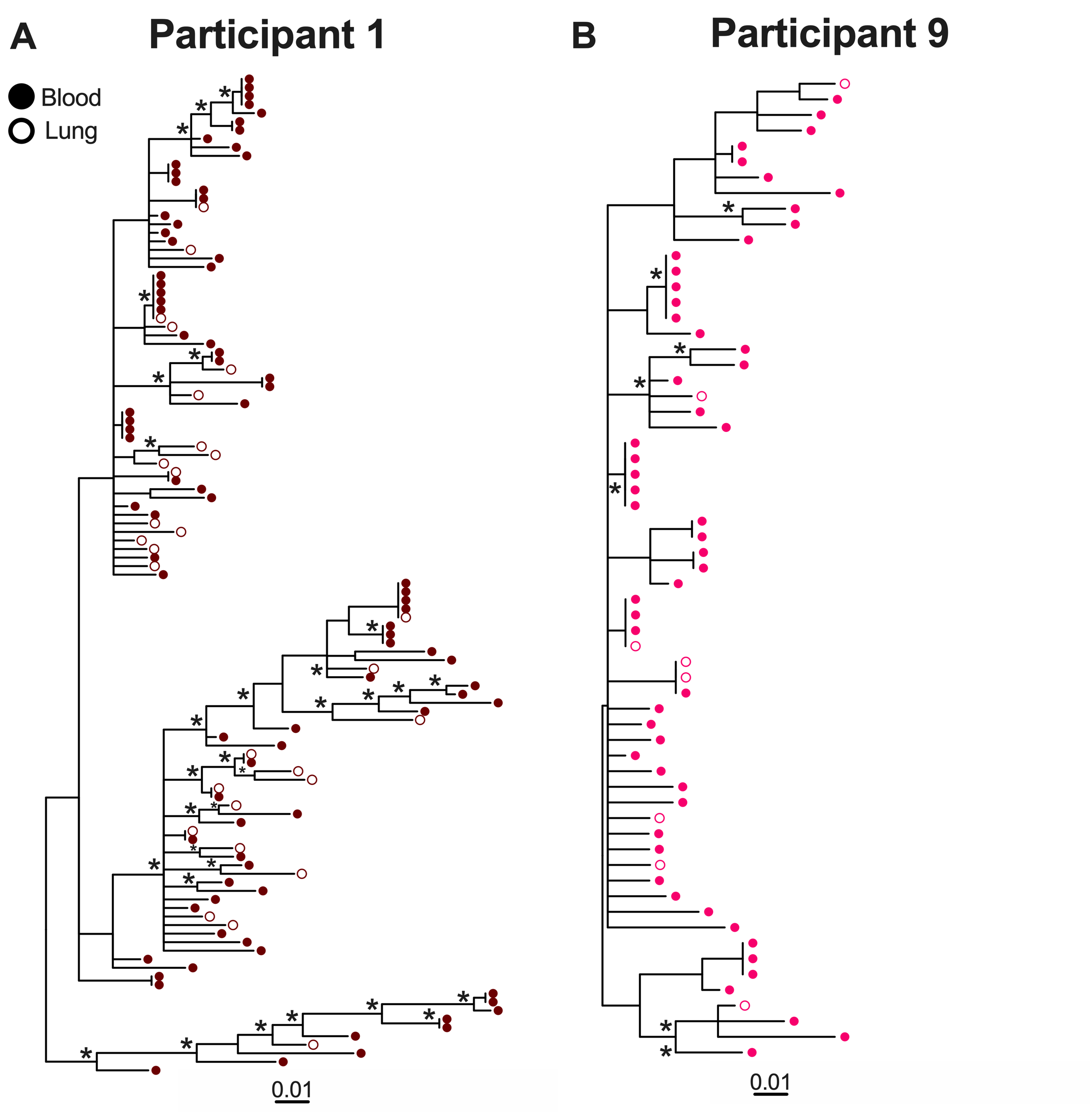

Supplement: S5 Fig — Legend as in S3 Fig. (TIF) [file ppat.1010613.s005.tif]

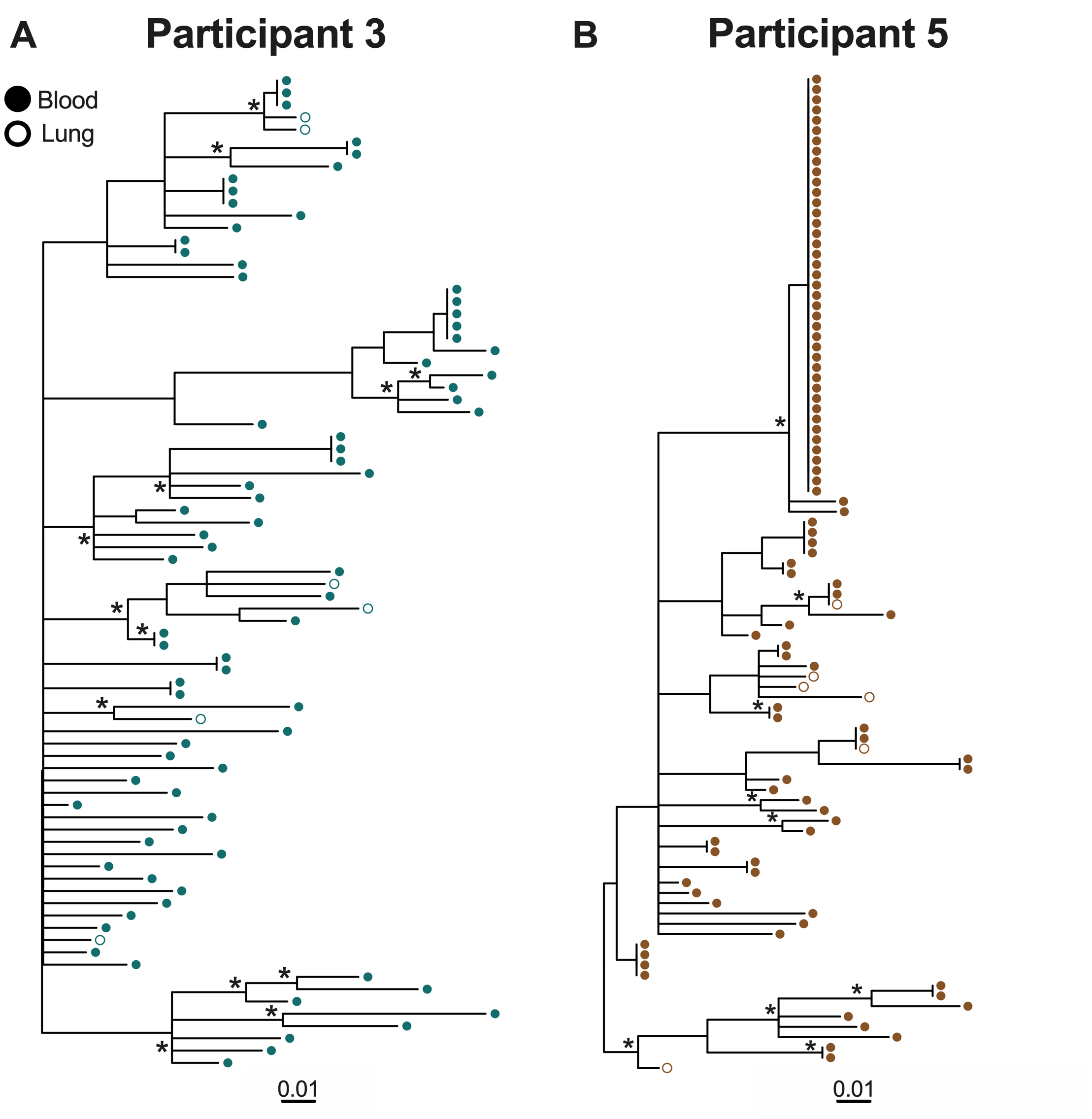

Supplement: S6 Fig — Legend as in S3 Fig. (TIF) [file ppat.1010613.s006.tif]

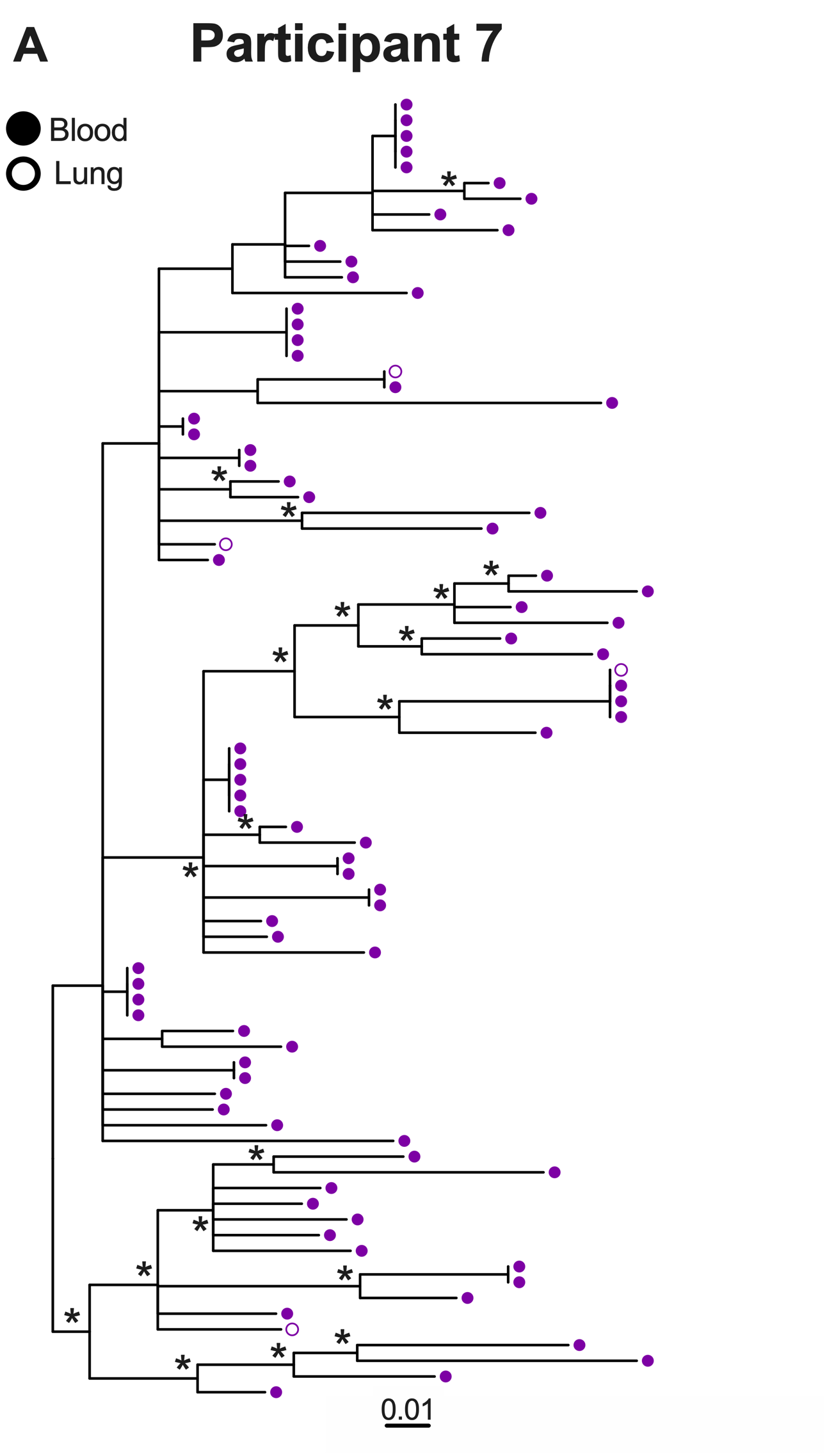

Supplement: S7 Fig — Legend as in S3 Fig. (TIF) [file ppat.1010613.s007.tif]

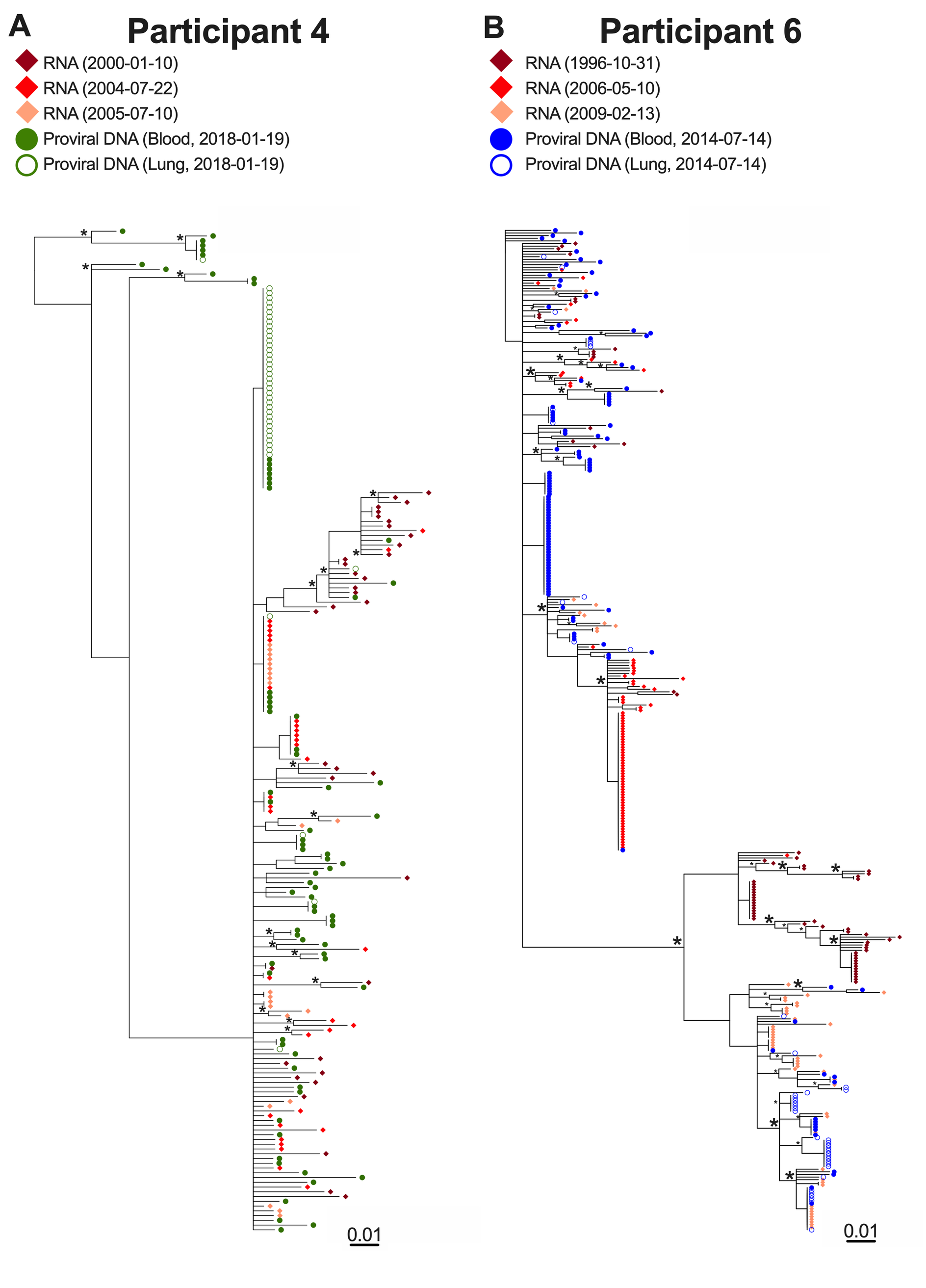

Supplement: S8 Fig — Consensus phylogenies derived from Bayesian inference of 7,500 outgroup rooted trees per participant. Filled circles denote blood sequences; open circles denote lung sequences; diamonds show plasma sequences. Asterisks identify nodes supported by posterior probabilities ≥70%. (TIF) [file ppat.1010613.s008.tif]

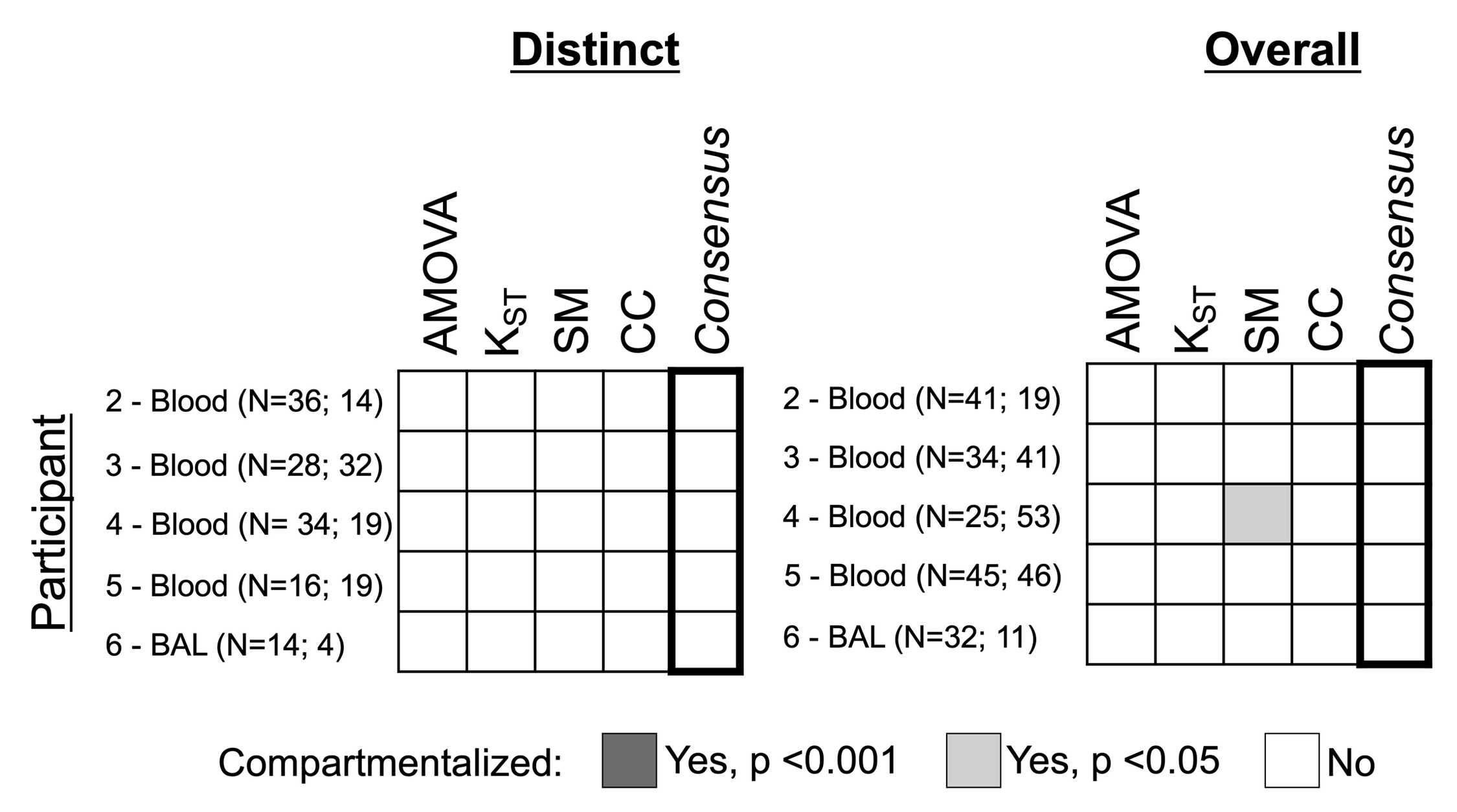

Supplement: S9 Fig — Summary of genetic compartmentalization results for the five participants for whom proviral sequences were collected from two independent aliquots of the same material, where a dataset is declared compartmentalized if at least one test per type (genetic distance vs. tree-based) gave a statistically significant result (see "consensus" column). The number of sequences recovered from each blood or lung aliquot is indicated in the participant label. Dark grey squares denote compartmentalization with p<0.001 (no test reached this threshold), light grey squares denote compartmentalization with p<0.05; white squares denote no compartmentalization. The "Distinct" panel summarizes compartmentalization results when limiting analysis to distinct sequences per compartment; the "Overall" panel summarizes results when all sequences are included. (TIF) [file ppat.1010613.s009.tif]
