# Supplementary material for: HIV proviral genetic diversity, compartmentalization and inferred dynamics in lung and blood during long-term suppressive antiretroviral therapy
Source: PLoS Pathog. 2022 Nov 4;18(11):e1010613. doi: 10.1371/journal.ppat.1010613 (PMC9668181; doi:10.1371/journal.ppat.1010613)
Supplement: S1 Table — (DOCX) [file ppat.1010613.s010.docx]

**S1 Table**: Best-fit models of nucleotide substitution

| **Participant ID** | **Model** |
| --- | --- |
| 1 | SYM+I+G |
| 2 | GTR+I+G |
| 3 | SYM+I+G |
| 4 | HKY+I+G (same for Fig 6)* |
| 5 | HKY+I+G |
| 6 | HKY+I+G (GTR+I+G for Fig 7)* |
| 7 | HKY+I+G |
| 8 | GTR+I+G |
| 9 | HKY+I |

SYM = Symmetrical model; GTR = General Time Reversible model; HKY = Hasegawa, Kishino and Yano model. Where applicable, models include Invariable site plus discrete Gamma distribution with four rate categories (+I+G) or Invariable sites (=I).

*For participants 4 and 6, ModelFinder and jModelTest returned the same models for the alignments of within-host proviral and plasma HIV RNA *nef* sequences (used in Figures 6, 7 and the trees inferred for the topological tests).
