# Supplementary material for: HIV proviral genetic diversity, compartmentalization and inferred dynamics in lung and blood during long-term suppressive antiretroviral therapy
Source: PLoS Pathog. 2022 Nov 4;18(11):e1010613. doi: 10.1371/journal.ppat.1010613 (PMC9668181; doi:10.1371/journal.ppat.1010613)
Supplement: Attachment — Submitted filename: Response to reviewers_13 Sep 2022.docx [file ppat.1010613.s012.docx]

Reviewer comments are in black. Our responses are in blue, *with text quoted from the revised manuscript in italics.*

**Part I - Summary**

Reviewer #1: Shahid et al examine viral diversity in the lung and blood during ART. This is an extremely interesting and understudied topic. Based on DNA-derived nef sequences from the blood and lungs during suppressive ART and RNA-derived sequences from the blood during viremia, the authors make a number of interesting observations including the following. First, they do not observe extensive compartmentalization in the lungs, suggesting that prior to ART, viral populations do not replicate extensively at this site independent of variants in the blood. Second, they observe identical nef sequences in the blood and lungs further illustrating mixing between these compartments. Finally, they observe a higher percentage of clonal sequences in the lungs than blood. Together these results are consistent with viral dynamics in the lungs possibly being driven by inflammatory processes generating migration of infected cells from the periphery to the lungs and clonal expansion in the lungs.

We thank the reviewer for the positive comments regarding the importance of our study and the interesting nature of our findings.

Unfortunately the study has a number of issues that limit the strength of these (and other) conclusions. One unsurprising issue is that the authors typically generated few sequences from the lungs and much larger numbers from the blood. As a result, it is difficult to define features of viral population in the lungs and assess compartmentalization. Second, some of their phylogenetic approaches may not be appropriate for the complex samples that they are analyzing. For participant 6 they constructed maximum likelihood trees whose root was intended to be the MRCA. However, because of the participants unusual suppression history and a lack of longitudinal pre-ART samples, their approach rooted the tree to sequences from an intermediate timepoint despite the tree containing RNA-derived sequences from a much earlier timepoint. This violates the assumption that the root is the MRCA of the population. These concerns make it challenging to generate conclusions about viral dynamics in the lungs.

We thank the reviewer for this feedback. The low number of sequences recovered from BAL is indeed a limitation of ours and prior studies of lung proviral diversity during ART, as these sequences are difficult to isolate. Unfortunately, no BAL remains for any participant so we cannot increase lung sequence N. As suggested by the editors, we have moved the three participants with the fewest lung sequences to the supporting information, and we now present all cohort-level analyses both including and excluding these participants.

We also acknowledge that the strategy that we used to root participant 6's phylogeny was unconventional and potentially subject to unknown biases. As described in detail below, we have changed our approach. Briefly, we now use the more standard approach of outgroup rooting for both participants 4 and 6, and then apply five published topological tests to further support the identified root locations as appropriate. This new approach changes the root location of participant 6 from what was originally presented, so that the sequences closest to the root are now from the persistent proviral pool, which is expected to contain at least some archived sequences.

Reviewer #2: Shahid et al. sequenced the nef genomic region of proviruses within cells from the lung and compared these nef sequences from the lung to proviral sequences from blood-derived cells in nine participants on long-term therapy. They found identical sequence expansions varied across participants and that 77% of the participants had identical proviral sequences from the lung and blood anatomic sites. The authors employed four methods for measuring compartmentalization of proviral sequences between the lung and blood anatomic sites and observed little evidence for lung-blood compartmentalization of proviral sequences in 5 out of the 9 participants. However, they did find large differences in the proviral dynamics as exhibited by the proportion of persisting proviruses that represented ancestral versus more recently-circulating viral sequences. This is a well-written and interesting study focusing on an understudied anatomic site, the lung, and provides important genetic analysis of the proviruses found in this site compared to the blood, However the are some limitations of the study which the authors should address and discuss.

We thank the reviewer for the positive comments on the quality and importance of our paper. Our revisions to address and discuss the limitations raised are detailed below.

Reviewer #3: Shahid and colleagues compare HIV proviral populations in blood and lung in individuals with viremia suppressed on long-term ART. The authors describe how their study differs from previous work and adds to our understanding of HIV persistence. They demonstrate modest compartmentalization in the lung of some people living with HIV that likely results from different frequencies of infected T cells clones and from infection of lung macrophages. The study supports the idea that, overall, measuring the HIV reservoir in blood is a good representation of virus that may be found in tissues such as lung (presented here).

We thank the reviewer for the accurate summary of our study.

Suggestions:

1) In the Author Summary, change last sentence from "Critically, the extent of within-host proviral diversity in blood correlated strongly with that in lung, indicating that despite inter-individual heterogeneity, blood is a strong indicator of proviral diversity elsewhere in the body" to "Critically, the extent of within-host proviral diversity in blood correlated strongly with that in lung, indicating that despite inter-individual heterogeneity, blood is a strong indicator of proviral diversity in the lung" since other tissues were not evaluated. Some studies have shown good evidence of compartmentalization in brain.

We have made this change throughout the manuscript. The author summary now reads (lines 61-62): *"Blood proviral diversity mirrored that in lung, indicating that strategies to limit the former (e.g. early ART) should also limit the latter".*

2) Table 1- 1) Change word "intact" to "nef". Intact infers full-length HIV sequencing. 2) Add estimated duration of infection prior to ART initiation

We have changed "intact" to "*nef*-intact" throughout the manuscript. Unfortunately, we do not have complete information on duration of infection prior to ART initiation, but we have added the information that we do have into Table 1. The revised table is shown below.

**Table 1.** **Participant information and HIV sampling details**

| **ID^a^** | **Sex** | **Duration of uncontrolled infection (years)** | **Years of ART prior to proviral sampling** | ***nef*-intact proviral lung sequences**  **Total N**  **(distinct N; %)** | ***nef*-intact proviral blood sequences**  **Total N**  **(distinct N; %)** | **pre-ART plasma HIV RNA *nef*-intact sequences^a^**  **Total N**  **(distinct N; %)** |
| --- | --- | --- | --- | --- | --- | --- |
| 1 | M | unknown | 8 | 29 (29; 100%) | 88 (64; 72%) | - |
| 2 | M | >9 | 17 | 31 (13; 42%) | 60 (50; 83%) | - |
| 3* | M | >19 | <1 | 6 (6; 100%) | 75 (60; 80%) | - |
| 4 | M | >13 | 18 | 42 (7; 17%) | 78 (53; 68%) | 91 (65; 71%) |
| 5* | M | unknown | 5 | 6 (6; 100%) | 91 (35; 38%) | - |
| 6 | M | >15 | 12 | 43 (18; 42%) | 126 (56; 44%) | 211 (99; 47%) |
| 7* | M | unknown | >10 | 4 (4; 100%) | 79 (57; 72%) | - |
| 8 | F | unknown | 9 | 14 (2; 14%) | 63 (58; 92%) | - |
| 9 | M | <1 | 3 | 8 (7; 88%) | 55 (40; 73%) | - |
| **Total** | |  |  | **183 (92; 50%)** | **715 (473; 66%)** | **302 (164; 54%)** |

^a^Participants 3, 5, and 7 yielded 10-fold fewer lung than blood proviral sequences. Within-host phylogenies for these participants are in the Supporting information (S1 and S2 Figs), and cohort-level analyses are performed both including and excluding these participants.

^b^Plasma HIV sequences were collected from three historic time points, including pre-ART, for each of participants 4 and 6. Historic plasma was not available for the other participants.

3) Remove the word "intact" through the text since it implies full-length HIV sequencing. Use “nef” or sub-genomic” instead throughout.

We have changed this wording to "*nef*-intact" throughout the manuscript.

4) Please review the statistics for the compartmentalization tests. It does not seem that they were corrected for multiple comparisons. For example, it is typical for the KST to be significant when the p value is <10-3 when applied to single-genome sequencing data to correct for sampling error of highly diverse HIV populations in vivo. I am not sure if the p value of <0.05 was used appropriately for these tests.

The reviewer is correct that our threshold for significance was p<0.05. Though the reviewer mentions studies that have used more stringent p-values for compartmentalization analyses, we and others have also previously used the standard p<0.05 when analyzing single-genome sequences for compartmentalization (*e.g.* Miller *et al*, J Virol, PMID: 31189714; Sturdevant *et al*, PloS Path, PMID: 23300446; Joseffson *et al*, PNAS, PMID: 24277811), even when inferring multiple phylogenies per dataset as was done presently (*e.g.* Jones *et al*, J Virol, PMID: 31776273). As the reviewer points out, it would be inappropriate if we tested 7,500 phylogenies per participant and then declared a dataset to be significant if ***any*** test returned a significant result. But, we require the ***mean*** p-value for all 7,500 phylogenies be <0.05 in order for it to be declared compartmentalized (and we provide the 95% HPD for transparency). We therefore respectfully request to leave our significance threshold as-is, but we have nevertheless amended the text to make this more clear (see lines 185-186). We have also amended Fig 5A to display the level of significance achieved by each test.

5) The statement in line 365 that claims that the data shows that clonal populations of infected cells are not present in all patients should be removed. When identical sequences are not found, it does not mean that clones of infected cells are not present since each of the sequences detected could be part of small cell clones that were detected only once at the level of sampling in the study.

We have changed this sentence to (lines 384-388):

*While this supports clonal expansion of reservoir cells as a common driver of HIV persistence during ART [14,15,57], it indicates that we cannot make generalizations regarding where clonally-expanded reservoir cells are more likely to be found, and also reveals that large clonally-expanded populations are not abundant in everyone at all times.*

6) The word "clades" should be changed to "variants" in line 390.

We have changed this to "sequences".

7) I suggest adding more discussion on how sampling affects the analyses. For example, if you had sampled sequences in the blood or lungs "twice", could occasional "compartmentalization" be seen in different donors just because few proviral sequences are obtained relative to all the infected cells in the body? I would also further discuss the effect of shallow sampling of the lungs, emphasizing that the large rakes of identical sequences in the lungs are likely very large infected cell clones or identical variants that infect lung macrophages.

We thank the reviewer for this comment. For five participants, we had sampled blood or lung sequences from two separate aliquots of the same sample, so we applied the full suite of compartmentalization tests to these two "batches" of data to investigate whether false-negative compartmentalization results can occur when re-sampling the same biological material more than once. We applied the same threshold for declaring compartmentalization as the main paper (requirement for p<0.05 on at least one of each type of test). As expected, we did not detect compartmentalization in any case where the same material was sampled more than once. For the reviewers' benefit, we summarize the full set of results in the below table.

| **Based on distinct sequences** | | | | | |
| --- | --- | --- | --- | --- | --- |
| **Participant** | **Sequence Ns^a^** | **K_ST_** ϕ **, p-value** | **AMOVA statistic, p-value** | **SM # migrations (mean, 95%HPD p-value)** | **CC statistic (mean, 95%HPD p-value)** |
| 2 | Blood (36, 14) | 0.02, 0.496 | 0, 0.423 | 13 (0.893, 0.776 - 0.999) | -0.008 (0.55, 0.230 - 0.832) |
| 3 | Blood (28, 32) | 0.01, 0.739 | 0, 0.535 | 19 (0.429, 0.086 - 0.76) | 0.007 (0.327, 0.043 - 0.610) |
| 4 | Blood (34, 19) | -0.004, 0.83 | 0.043, 0.087 | 14 (0.393, 0.045 - 0.815) | 0.008 (0.390, 0.045 - 0.815) |
| 5 | Blood (16, 19) | 0.024, 0.619 | 0, 0.794 | 14 (0.920, 0.666 - 0.999) | -0.03 (0.828, 0.558 - 0.999) |
| 6 | BAL (14, 4) | 0.068, 0.316 | 0.04, 0.258 | 4 (0.842, 0.190 - 0.999) | -0.02 (0.541, 0.239 - 0.810) |
| **Based on overall sequences** | | | | | |
| **Participant** | **Sequence Ns^a^** | **K_ST_** ϕ **, p-value** | **AMOVA statistic, p-value** | **SM # migrations (mean, 95%HPD p-value)** | **CC statistic (mean, 95%HPD p-value)** |
| 2 | Blood (41,19) | 0.017, 0.443 | 0.017, 0.186 | 16 (0.584, 0.419 - 0.764) | -0.002 (0.508, 0.136 - 0.832) |
| 3 | Blood (34, 41) | 0.01, 0.524 | 0, 0.449 | 23 (0.345, 0.028 - 0.618) | 0.01 (0.187, 0.012 - 0.382) |
| 4 | Blood (25, 53) | -0.020, 0.952 | 0.052, 0.052 | 18 (0.033, 0 - 0.08) | -0.02 (0.727, 0.305 - 1) |
| 5 | Blood (45, 46) | 0.009, 0.537 | 0, 0.661 | 36 (0.51, 0.269 - 0.733) | -0.01 (0.479, 0.209 - 0.747) |
| 6 | BAL (32, 11) | 0.0126, 0.486 | 0, 0.894 | 11 (0.874, 0.289 - 0.999) | -0.01, (0.593, 0.162 - 0.938) |

^a^ Sequence numbers recovered from each sample aliquot

The revised manuscript summarizes the results of this analysis (on lines 449-453) and presents the overall findings in the supporting information S9 Fig, shown at right.

We have also expanded on the potential implications of shallow sampling of the lungs (lines 447-449): *"low lung sequence numbers limited our ability to estimate proviral parameters (% distinct, nef-intact, etc) and detect compartmentalization in a subset of participants"*.

And, we have added the reviewer's suggested comment regarding the large numbers of identical sequences in some individuals (lines 478-481): "*Our isolation of large numbers of identical sequences from the lungs of some individuals, strongly suggestive of clonal expansion, suggests that these are derived from CD4^+^ T-cells (as macrophages are non-dividing [67]), though we cannot rule out that these are nef-identical variants that infected large numbers of lung macrophages".*

8) I would remove the lines connecting the points in Figure 1B and, instead, add a bar at the median values since, I think, that is what the p value is referring.

We apologize for the lack of clarity. The p-value reports the results of	a paired test that compares each participant's blood and lung values, so the lines are informative as they connect each participant's blood and	lung samples. We have changed the color of the connecting lines to match each participant's color, and we now also state the name of the test (new Figure 1B shown at right).

9) Line 150: “…highest likelihood phylogeny from among 75,000 within-host reconstructions is shown.” Some justification should to be added for why the authors select the highest likelihood topology. Typically, the consensus tree from the Bayesian analysis is used. Selecting the highest likelihood could introduce bias as it doesn’t represent the Maximum clade credibility (>95%) of the 75,000 within-host reconstructions. Same comment for Line 519.

We appreciate this question. We selected a representative output	tree for visual display (rather than the consensus tree)	for ease of interpretation. Due to the inherent uncertainty	in phylogenetic reconstruction, particularly on within-host	datasets, consensus trees typically contain polytomies and	unresolved subclades, which can make them difficult	to interpret (indeed, the reason we inferred 7,500 trees per	participant was to overcome the inherent uncertainty	associated with inferring only one tree per dataset). The	displayed tree is meant to be a representative visual only	(though with highly-supported branches identified for	transparency), while the results shown below it report	the results from all 7,500 trees. Nevertheless, we agree	that showing the consensus trees is important for transparency, so these are now shown in Supplemental information	(S3 Fig to S8 Fig). Two representative consensus trees are	shown at right (from S3 Fig).

10) Lines 382-384: “Overall, our results indicate that compartmentalization is generally limited, of modest magnitude, and when present it is often due to differential distributions of clonally-expanded populations.” A discussion of the pros/cons for the statistical tests used might be useful.

Thank-you for this helpful comment. We have added a discussion of the pros/cons of the different test as follows (lines 488-500):

*“….A brief discussion of the advantages and limitations of each test is also useful. The tree-based methods, SM [48] and CC [49], have the advantage of leveraging the evolutionary relationships among the sequences as informed by the tree, and generally have more power to detect compartmentalization [45]. These approaches however assume a high level of certainty in the underlying phylogeny, which is often not the case for within-host trees. To mitigate this, we integrated these tests over a Bayesian sampling [70] of 7,500 trees (the inherent uncertainty in phylogenetic inference can be seen in the consensus trees; see Supporting information). It is also important to note that the tree-based tests consider topology (branching patterns) only, not branch lengths (long branches would thus convey the same information as shorter ones, despite describing a greater genetic distance), and that SM relies on a migration model whereas CC merely compares the correlation of the distance between tips. The genetic distance-based tests, AMOVA [46] and K_ST_ [47], have the advantage of being able to provide results even when a phylogeny is difficult to resolve [37]”.*

11) Line 435: A parenthesis needs to be added.

We have made this change.

12) Line 502: Did ModelFinder demonstrate any uniquely different models for each PID, or was it consistent? If so, might be worth including.

The best fitting models for each participant's within-host dataset are now shown in S1 Table (reproduced at right). Note that jModelTest was used for model selection for Bayesian phylogenies, while ModelFinder was used for maximum-likelihood phylogenies.

13) Line 506: Where ModelFinder and jModelTest in agreement?

As ModelFinder was used to select the best models for the maximum-likelihood phylogenies, while jModelTest was used to select the best models for the within-host Bayesian analyses, there were only two instances where both tests were applied to the same data. These were the combined proviral and historic HIV RNA sequence datasets for participants 4 and 6 (*i.e.* the Bayesian trees shown in Fig 6 and 7 an the corresponding trees for the topological tests). In both cases, there was complete agreement between tests: for participant 4, both models identified HKY+I+G for all analyses performed, while for participant 6's phylogeny that included proviral and HIV RNA sequences, both models identified GTR+I+G. This information is in S1 table (shown above)

14) Line 509-510: Without the statement, is it to be assumed that no clock was enforced? But if it was, the arithmetic/harmonic means of the null and +clock should be included as well as an explanation for its use. Since the Bayesian analysis was not conducted in BEAST, it appears that no clock was enforced.

This is correct. As the trees were made in MrBayes, where the goal was to infer 7,500 phylogenies from each within-host dataset, that were unconstrained by any molecular clock assumptions, no clock was enforced. This information has been added to the methods on line 566.

15) Line 542-547: How does the patristic distances differ from an APD calculation? The authors report the percent of distinct sequences and genetic distance within each compartment, but the explanation of diversity between

The last part of this comment was cut off, but we interpret it to be a question about our diversity metrics. Patristic distances and average pairwise distances (APD) are both pairwise distance metrics, but patristic distances refer explicitly to phylogenetically-inferred pairwise (*i.e.* tip-to-tip) distances, whereas APD are calculated directly from sequence alignments using genetic distance metrics. We have defined the term "patristic" more clearly where it first appears (lines 147-148).

**Part II – Major Issues: Key Experiments Required for Acceptance**

Reviewer #1: 1) There are a number of issues with the compartmentalization analyses that should be considered.
Uneven and small sample sizes greatly reduce the power and accuracy of compartmentalization analyses (Zarate et al 2007). In this study 4 of 9 participants had fewer than 10 sequences from the lungs and an average of 75 sequences from the blood. The small number of lung sequences and the very large number of blood sequences makes it impossible to accurately assess compartmentalization for these individuals. This problem is worse when the analysis is restricted to distinct sequences in which case 6 of 9 participants had fewer than 10 sequences from the lungs and an average of 48 sequences from the blood. The primary finding of the paper is that there isn't evidence of major compartmentalization in the lungs, however, the limited sampling reduces the power of the analyses.

The low number of lung sequences recovered from some participants is indeed a limitation, and we wish that we could have isolated more sequences from BAL. Recovery of these sequences however is difficult, and other studies performed on ART-suppressed individuals have featured the same limitation (*e.g.* Bozzi *et al,* Sci Adv*,* PMID: 31579817 recovered lung sequences from only two participants, one of which had only eight sequences; Rose *et al*, J Virol, PMID: 27466425 recovered lung sequences from only three participants, one of which had only three *nef* sequences). As suggested by the editors, we have relocated the three participants with 10-fold fewer lung than blood sequences to the supplement, to focus the compartmentalization results on the participants with larger numbers of lung sequences. In addition, we revised our text to further highlight the magnitude of the test statistic itself, which provides a measure of the *extent* of compartmentalization (rather than focusing on the p-value). This is important because the test statistics for the two datasets that showed compartmentalization in the "distinct" analysis were of low magnitude, indicating that compartmentalization, though statistically significant, was actually quite weak. We now also directly state that the low lung sequence numbers limited our ability to detect compartmentalization in some individuals (see lines 447-449) and we have revised our interpretations throughout (*e.g.* lines 390-406):

*“We also investigated the extent of blood-lung genetic compartmentalization, and whether this tends to manifest as distinct viral lineages across sites, or simply as differential proportions of clonally-expanded populations. With respect to the former, no dataset was so genetically compartmentalized that it was visually apparent in a phylogeny, but when we applied formal tests two participants (2 and 6) showed modest yet statistically significant evidence of distinct viral lineages across sites. The type of compartmentalization that is attributable to differential frequencies of identical sequences across sites was detected in two additional participants (4 and 8), where the magnitude was generally stronger. Compartmentalization was not detected in the remainder of participants, though for three of these (3, 5 and 7) insufficient recovery of lung sequences limited our power to detect it. Despite this, our results nevertheless suggest that "true" blood-lung proviral compartmentalization on ART (defined as the presence of genetically distinct viral variants) is not the norm, and when present it is generally of modest magnitude. The type of proviral genetic compartmentalization that is due to differential clonal expansion is more common, but still by no means universal. These observations are consistent with studies of viral diversity in lung [37, 38], female genital tract [42], testes [41], and lymph nodes [58]”.*

Even though the limited lung sequence numbers limit our power to detect compartmentalization in some participants, these sequences are nevertheless valuable to our understanding of on-ART lung proviral diversity. For example, if we had recovered four lung sequences from a participant and these were all identical to one another (and matched a large clone in blood), this would suggest something very different about lung proviral composition than if all four sequences were genetically divergent from another and distinct from blood sequences. The very strong correlation between blood and lung proviral diversity is another example of a useful biological insight that can be gleaned despite limited lung sampling in some individuals, though we now present the results of these analyses both including and excluding the three participants with the fewest lung sequences (new figs 5B, C at right).

• Given Zarate's observation that distance and tree-based methods have different strengths and weaknesses, a more conservative approach would be to declare compartmentalization significant if at least one test of each type is significant.

We have revised our manuscript to now require at least one test *of each type* to meet the significance threshold in order to declare a dataset to be compartmentalized (lines 163-164; 614-615; Fig 5A legend).

• The rationale behind the "distinct" analysis, i.e. "where any sequence present in both lung and blood was represented once per compartment" is unclear to me. This suggests that if multiple identical sequences were observed in one compartment all of those sequences would be included in the analysis, but if they were present in two compartment only a single sequence would be included from each. Is that correct? By analyzing multiple identical sequences within but not across the compartment, wouldn't that bias the analyses toward identifying compartmentalized lineages?

We apologize that this was not clear. The purpose of the "distinct" analysis was to test for compartmentalization *without* confounding by clonal expansion, so the inclusion of duplicate sequences would have indeed biased the analysis as the reviewer suggests. The analysis **did not** include duplicate sequences within a compartment. What the original text was attempting to explain was how we treated cases where the same sequence was present in both compartments (if this happened, it was included once per compartment). But, this was confusing, and the text now simply describes the "distinct" analysis as being restricted to *"only distinct nef sequences per compartment"* (line 187).

2) I understand the general rooting approach that the authors used for their trees containing plasma RNA, however, I'm not sure how intermittent suppression impacts this approach. The authors indicate that in regard to participant 6, "We therefore rooted each of the participant's 7,500 phylogenies at the location that maximized the correlation between root-to-tip distances and sampling time of plasma sequences collected in 2006 and 2009." However, the tree also contains pre-ART sequences from 1996. In addition, the reservoir is likely to be very diverse and all of the reservoir may not reactivate at the same time. Could this artificially increase the amount of diversity that accumulated between 2006 and 2009 and over-estimate the rate of evolution? It would be nice to include a more thorough discussion of this analysis and the impact that intermittent suppression may have on it.

We acknowledge that the rooting strategy originally used for this participant's dataset was unconventional and possibly subjected to unknown biases. We now use the more conventional approach of outgroup rooting for both participant 4 and 6's datasets, where we further undertake topology tests to support this root placement. Details of this new analysis are described in the response to the reviewer's next point.

3) "Assuming that the root represents the MRCA of the sequences collected, we can make some inferences about proviral dynamics." I don't see how this can be true. In figure 8 the root to tip distance was higher for samples collected in 1996 than samples collected in 2006. Thus the root can't be the MRCA of all the sequences in the tree. This approach is inappropriate for this dataset and causes the tree to misrepresent the within host evolutionary dynamics. The authors state " This strategy works because evolving within-host HIV populations exhibit increasing divergence from the root over time." However, the oldest sequences in the tree are not at the root. Given these concerns it is unclear that any conclusions can be made from the trees in figures 8 and 9. The authors should consider an alternative approach to analyses of sequences from these participants.

We appreciate this comment, and we have changed our approach. We now use outgroup rooting for both participants 4 and 6, and then apply five topological tests to further support the identified root location. While this approach does not substantially alter the original root placement (or interpretation) for participant 4, it does for participant 6. Now, the closest sequences to participant 6's root are from the persistent proviral pool sampled on ART, which is expected to contain at least some archived sequences (figure at right; on-ART proviruses are blue, pre-ART plasma sequences are maroon). This root location is likely more in line with the reviewer's expectation.

We now describe the rooting approach and topological testing on lines 580-591:

*"For participants 4 and 6, for whom pre-ART plasma HIV RNA sequences were available, MCMC runs were additionally performed on alignments of proviral (blood-lung) and plasma HIV RNA sequences using the best-fitting substitution model for each dataset as listed in S1 Table. Trees were rooted using an outgroup. As the ideal outgroup is distantly (but not too distantly) related to the ingroup, we rooted each participant's phylogeny using a sequence from the most closely-related participant in our study (see Fig 1C) - namely, participant 4's phylogeny was rooted using the closest sequence from participant 1, whereas participant 6's phylogeny was rooted using the closest sequence from participant 7. Outgroup rooting was performed using the evolutionary placement algorithm in RAxML, implemented with a custom script in R [83], after which the branch leading to the outgroup was pruned off. Consensus trees were generated from outgroup rooted trees using the consensus and consensus.edges function using ape and phytools package in R.*

*Topology testing of phylogenies*

*We inferred phylogenies in a maximum-likelihood framework using IQTree v1.6.1 [84] from participant 4 and participant 6’s sequences using the best-fitting nucleotide substitution model selected by ModelFinder (HKY+F+I+G4 and GTR+F+I+G4 respectively). For each participant, one phylogeny was inferred under the constraint that plasma HIV RNA sequences from the earliest time point were separated from the plasma sequences sampled at later time points (though not necessarily separated from proviral sequences). This constrained phylogeny was compared with an unconstrained phylogeny through comparison of the tree likelihoods using IQTree’s ‘-au’ command, which performs five topology tests: the resampling of estimated log-likelihoods (RELL) test [52], the Kishino-Hasegawa (KH) test [53], the Shimodaira-Hasegawa (SH) test [54], the approximately unbiased (AU) test [55], and the Expected Likelihood Weights (ELW) test [56]".*

Regarding the reviewer's general statement that the oldest sequences should be at the tree's root, we agree completely. However, since the present trees feature historic plasma HIV RNA sequences as well as sequences archived in the reservoir, we would not necessarily expect the oldest sequences to be plasma sequences that were sampled the longest ago. Instead, it is possible that some of the proviruses sampled on ART may represent the genetically oldest in the dataset (and thus closest to the root), if they had integrated before the earliest plasma sampling date. Indeed, the trees are consistent with this expectation: for both participants 4 and 6, the closest sequences to the root are proviruses sampled from the reservoir. Importantly, we now perform topology tests to assess whether constraining the earliest plasma sequences to be separated from the plasma sequences sampled at later time points results in an improved topology. It did not in either case, suggesting that sequences from the earliest sampling time point do not constitute a founder lineage that gave rise to the remainder of the participants’ sequences, and therefore may not adequately inform a temporal signal.

Participant 6's new rooted tree is now described as follows (lines 298-310):

*“Notably, the proviruses sampled after almost 18 years on ART interspersed throughout the whole phylogeny and represented all of the sequences near the root, which was far deeper in the tree than any sampled plasma HIV RNA sequence, suggesting that by far the most ancestral sequences sampled from this participant were on-ART proviruses. To further support this root placement, we compared an unconstrained maximum likelihood phylogeny to one where the sequences from the earliest plasma sampling (in February 2000) were constrained to be separated from the sequences sampled from plasma in 2004 and 2005, but not necessarily from the proviral sequences. The unconstrained tree's log likelihood was 44 units higher than the constrained one, yielding p-values < 0.05 in support of the unconstrained trees on all five topology tests performed [52-56]. This suggests that the plasma sequences sampled in Feb 2000, despite being sampled the earliest in calendar time, do not constitute an ancestral lineage that gave rise to the remainder of the participants’ sequences, thus supporting the outgroup-guided root placement”.*

4) The authors should clarify the goals of examining compartmentalization in this cohort. With a few exceptions, studies have not found evidence of ongoing viral replication during ART, thus compartmentalization analyses in people on ART are typically examining whether distinct populations were established in a compartment prior to ART. The authors indicate that participant 9 initiated ART soon after diagnosis thus limiting their ability to assess viral replication in the lungs pre-ART. Another interesting question is whether HIV-infected cells migrate between the blood and tissues during ART. However, addressing this hypothesis may require knowledge of the population in the lungs before ART. The authors should specify the hypotheses that they are testing and the type of data that can be used to address those hypotheses.

We thank the reviewer for this suggestion. Our hypothesis is the very fundamental one of whether proviral populations persisting in blood and lung during long-term ART are genetically distinctive from one another (versus the null hypothesis that they are drawn from a single population). We have amended the introduction to state this directly, as follows (lines 102-110)

*"Our goal was to characterize the diversity of proviral populations persisting in lung during ART, and to assess whether these tend to be genetically compartmentalized from those in blood, and if so in what way. That is, we asked whether blood and lung typically harbour truly genetically distinctive proviral populations (which, if so, would likely have been established prior to ART, since no or negligible viral replication occurs during this period [6, 40]), or whether these sites tend to differ only in terms of the number and distribution of clonally-expanded reservoir cells [41, 42], or whether there is generally limited evidence to support blood-lung compartmentalization of any kind".*

As the reviewer points out, any genetic differences between populations could not have arisen due to ongoing viral evolution during ART, since no (or negligible) viral replication occurs during this period. Since our datasets comprise blood and lung proviral sequences sampled on ART at a single time point however, our ability to infer the *mechanism* of compartmentalization is somewhat limited. We now expand on this limitation, and possible interpretations, in the discussion on lines 453-464:

*“Since blood and lung proviruses were sampled at a single time point on ART, we cannot establish the mechanism of compartmentalization when it was detected. That is, distinctive HIV populations detected in blood and lung were likely already established prior to ART (since HIV replication is negligible or non-existent during ART [6, 40]), but we cannot exclude the possibility that proviral populations pre-ART were similar, but that certain proviral lineages subsequently decayed at differential rates between sites, yielding distinctive populations when sampled years later. Similarly, datasets that exhibited the "differential clonal expansion" type of compartmentalization are likely the result of dynamic changes in clonal expansion during ART [13], but we cannot rule out the existence of imbalanced clonally-expanded populations prior to ART, nor can we conclusively identify the site where the clonal expansion occurred (e.g. clones could have arisen in one compartment, migrated to another, then largely disappeared from the former)”.*

5) Given the small number of lung sequences obtained for many of the participants, it is difficult to accurately estimate parameters about those sequences (percent distinct, hypermutated, intact, etc). As a result, it's difficult to make generalizations about differences across participants (See Fig 1b) and compartments (See 7b and 7c). This should be mentioned whenever discussing differences.

Figs 1B, 5B and 5C now report the overall statistics for the whole dataset, as well as after removal of the three participants with the fewest lung sequences (See new Fig 5B, C on page 7 of this document). Our discussion also now directly states: *"low lung sequence numbers limited our ability to estimate proviral parameters (% distinct, nef-intact, etc) and detect compartmentalization in a subset of participants"* (lines 447-449).

6) Is it appropriate to compare divergence from the root in participants 4 and 6 despite the fact that one was rooted using HXB2 and the other using RNA derived sequences from that participant?

Although both participants are now outgroup rooted, this comment is still relevant. After rooting, the branch leading to the outgroup is pruned off, so that the resulting root reflects the MRCA of the participant's dataset, not the root that is ancestral to both the outgroup and the participant's dataset (which would indeed prevent comparisons of trees that were rooted different ways). We have clarified this in the methods on lines 587-589: "*Outgroup rooting was performed using the evolutionary placement algorithm in RAxML, implemented with a custom script in R [82], after which the branch leading to the outgroup was pruned off".*

7) For clarity, please specify that you are looking at nef sequences in table 1 (e.g. "intact nef DNA sequences from the lung ")

We now refer to sequences as "*nef*-intact" throughout the manuscript - see new Table 1 on p. 2 of this document.

Reviewer #2: 1) The authors state several limitations of their study in their discussion section of the manuscript but they do not address whether the limited number of proviral sequences from the lung compartment for 4 participants (<10 sequences in total) affected the accuracy of the methods used to determine compartmentalization between the lung and blood anatomic sites. For many compartmentalization methods if one site has approximately 10-fold more sequences (ie the blood-derived proviral sequences), this can affect the accuracy of the methods used to calculate compartmentalization. The authors should discuss in their results section whether the disparity in the number of proviral sequences between the lung and blood compartments could affect their results. If so, how did they address this issue?

Three of the study participants (3, 5 and 7) who had more than tenfold more blood than lung sequences have been explicitly identified as such (*e.g.* lines 121-123: *"Participants 3, 5, and 7 yielded 10-fold fewer lung than blood proviral sequences, which limits our ability to detect compartmentalization and possibly other within-host proviral genetic features"*). This limitation is also re-emphasized in the discussion: *"low lung sequence numbers limited our ability to estimate proviral parameters (% distinct, nef-intact, etc) and detect compartmentalization in a subset of participants"* (lines 447-449). Moreover, and as described above, these three participants have been relocated to the supplement and all cohort-level analyses (Fig 1B, 5C, 5D) are now presented both including and excluding these participants.

2) An elegant study of the proviral diversity in context of pre-ART viral populations was presented for two participants. However, in this section the comparison of the pre-ART and treatment interruption plasma-derived sequences to the proviral sequences in the lung is not described in-depth. For each participant, the authors should add a small section as to how the plasma-derived RNA sequences compared to the lung proviral sequences. Is there any evidence or indication as to when the lung anatomic site was seeded? Was this site seeded prior to therapy initiation or during the treatment interruption phase?

We thank the reviewer for this suggestion, and have revised both sections accordingly. For participant 4, lines 319-330 now read:

*“Overall, these observations suggest that, while participant 4's replication-competent reservoir largely dates to around the time of ART initiation (as evidenced by the similar root-to-tip divergence measurements of the pre-ART sequences circulating in 2000 to those that rebounded in plasma in both 2004 and 2005), their overall proviral pool, that would also include defective proviruses, features sequences that are far more ancestral than this (as evidenced by the abundance of proviral sequences close to the root and the complete lack of plasma sequences within these ancestral subclades). The data also suggest that the 2004 and 2005 viremia rebound events re-seeded the reservoir to some extent. This is evidenced by numerous large subclades comprising blood and/or lung proviruses isolated in 2018 that were genetically identical to 2004 and/or 2005 plasma rebound viruses. By contrast, we isolated only one provirus that was genetically identical to a sequence that circulated in plasma just prior to ART initiation (Fig 6B, near the bottom of the tree)”.*

For participant 6, lines 363-375 now read:

*“Like participant 4, participant 6's phylogeny also suggests that these rebound plasma viruses re-seeded the reservoir. This is evidenced by numerous subclades of blood and lung proviruses isolated in 2014 that were genetically identical to sequences isolated from plasma in 2009 (near bottom of tree). A 2014 proviral sequence identical to a 2006 rebound virus, that was isolated 52 times from plasma, also supports this (middle of tree). By contrast, we did not isolate any proviruses that were genetically identical to any plasma viruses that circulated in 1996 pre-ART. Also of note, the sampled proviruses were somewhat less diverse and divergent than the 1996 pre-ART sequences (Fig 7C and D), largely because the latter featured a distinct subclade, that was quite divergent from the root, that contained only a single sequence from one other timepoint, a 2006 plasma sequence (about two-thirds down the tree). This suggests that representatives of this clade, which were abundant in plasma just prior to ART initiation, did not persist in abundance in the reservoir”.*

3) The authors found that the diversity of distinct proviral sequences recovered from blood correlated significantly with the diversity of distinct sequences recovered from lung for these participants. Although this was calculated using two methods, if the number of distinct sequences falls below 5, this can affect the accuracy of diversity calculations. Did any of the participants have less than 5 distinct sequences from the lung? If so, the authors should graph their data with and without these participants to determine if participants with low numbers of distinct proviral sequences from the lung affects the correlation of distinct proviral sequences between the lung and blood compartments.

The revised graph now flags the three participants whose data have been relocated to the supplement due to limited number of lung sequences, and the statistics are presented overall as well as excluding these three participants - please see new Figure on page 7 of this document. The correlations (in terms of Spearman's rho values) remain strong regardless.

4) In this study the authors sequence the nef region of pre-therapy and treatment interruption plasma-derived RNA. A recent full-length HIV RNA study has revealed that plasma-derived sequences are not all intact and that up to 50% are defective. The authors should describe how sequencing the nef region of HIV RNA may be a limitation and how this could affect their findings especially if this does not always represent a full-length RNA sequence or replication-competent virus.

Thank-you for this suggestion, which has now been incorporated into the discussion of limitations (and the suggested reference cited), lines 467-468: *"In fact, even subgenomic plasma HIV RNA sequences may not necessarily be derived from intact viral genomes, as recently demonstrated [Fisher et al*, *J Virol, PMID:* 35201897]**Part III – Minor Issues: Editorial and Data Presentation Modifications**

Reviewer #1: 1) The duration of untreated infection should be designated in Table 1.

As mentioned above, we have revised Table 1 to include estimated duration of untreated infection for the participants for whom this information is available (see page 2 of this document).

2) It's interesting that some participants primarily had clonal viruses in their lungs at the on-ART timepoint. Is there any evidence that they had a respiratory infection or other illness that could drive clonal expansion?

Two participants (8 and 9) had COPD, though the remainder did not have any document overt respiratory symptoms or lung disease. This information is in the methods (lines 526-528). Unfortunately however, we have no information on acute respiratory infection history.

Reviewer #2: This is only semantics but when referring to intact proviral sequences please change this to nef-intact proviral sequences.

We have made this change throughout.
